# Non-invasive brain stimulation for improving gait, balance, and lower limbs motor function in stroke

**DOI:** 10.1186/s12984-022-01062-y

**Published:** 2022-08-03

**Authors:** Jitka Veldema, Alireza Gharabaghi

**Affiliations:** 1 Department of Sport Science, Bielefeld University, 33 501 Bielefeld, Germany; 2 Institute for Neuromodulation and Neurotechnology, University Hospital and University of Tübingen, Tübingen, Germany

**Keywords:** rTMS, tDCS, tACS, tsDCS, Stroke, Gait, Balance, Lower limb function

## Abstract

**Objectives:**

This systematic review and meta-analysis aim to summarize and analyze the available evidence of non-invasive brain stimulation/spinal cord stimulation on gait, balance and/or lower limb motor recovery in stroke patients.

**Methods:**

The PubMed database was searched from its inception through to 31/03/2021 for randomized controlled trials investigating repetitive transcranial magnetic stimulation or transcranial/trans-spinal direct current/alternating current stimulation for improving gait, balance and/or lower limb motor function in stroke patients.

**Results:**

Overall, 25 appropriate studies (including 657 stroke subjects) were found. The data indicates that non-invasive brain stimulation/spinal cord stimulation is effective in supporting recovery. However, the effects are inhomogeneous across studies: (1) transcranial/trans-spinal direct current/alternating current stimulation induce greater effects than repetitive transcranial magnetic stimulation, and (2) bilateral application of non-invasive brain stimulation is superior to unilateral stimulation.

**Conclusions:**

The current evidence encourages further research and suggests that more individualized approaches are necessary for increasing effect sizes in stroke patients.

## Introduction

Each year, approximately 795,000 people experience a new or recurrent stroke [1]. Walking and balance disturbances are common post-stroke complications, affecting about two-thirds of stroke survivors [2]. These deficits are associated with worsened quality of life, impeded community reintegration [3], and an increased risk of falling [4]. The ability to walk independently is the most common rehabilitation goal after stroke [5]. However, about 50% of stroke survivors suffer from an impaired walking ability 6 months after current standard of care [2]. Other interventions are therefore needed to improve recovery. Thus, the development of innovative therapeutical strategies for improving balance and walking ability is one of the top research priorities in stroke rehabilitation [6]. Non-invasive neuromodulation methods such as repetitive transcranial magnetic stimulation (rTMS), transcranial direct/alternating current stimulation (tDCS/tACS) and trans-spinal direct current stimulation (tsDCS) can modulate neural processing and have thus the potential to counteract maladaptive neural plasticity after stroke and contribute to a better recovery [7, 8].

### Neural background of walking and balance

Neuroimaging studies have shown that walking and balance are complex sensorimotor functions controlled by integrated cortical, subcortical, and spinal networks [9–11]. A single photon emission computed tomography study demonstrates bilateral activation within the primary sensorimotor area, supplementary motor area, basal ganglia as well as within the visual cortex, cerebellar vermis, and part of the left lower temporal lobe during walking [12]. Similarly, a positron emission tomography study shows a bilateral increase of cerebral blood flow within the primary sensory cortex, primary motor cortex and supplementary motor cortex as well as within the anterior part of the cerebellum during active and passive bipedal movement [13]. A recent meta-analysis indicates a key role of the brainstem, cerebellum, basal ganglia, thalamus, and several cortical regions during postural control [9]. Accordingly, another meta-analysis shows that the cerebellum, basal ganglia, thalamus, hippocampus, inferior parietal cortex, and frontal lobe regions are involved during balance tasks [14]. Importantly, the available data indicates that also a spinal network may be involved in postural balance and gait control [15]. E.g., multiple studies demonstrate that balance training induces suppression of H-reflexes [16]. Thus, it is conceivable that the application of non-invasive brain stimulation over several cortical regions as well as over the cerebellum, the brainstem and the spinal cord may be effective in the modulation of walking, balance and/or lower limbs motor function.

### Stroke-induced changes of neural control during walking

Up to now, different neuroimaging techniques have been used to investigate the neural mechanism of walking disability and walking recovery in stroke patients. A large part of the available data demonstrates a stroke-induced disinhibition of the contralesional hemisphere with a shift of the between-hemispheric balance to the detriment of the affected hemisphere, as well as a correlation between normalization of neural processing and favorable motor recovery [17–21]. A diffusion tensor MRI demonstrates a between-hemispheric asymmetry in fractional anisotropy of the posterior limb of the internal capsule [18]. The shift of balance towards the non-lesioned hemisphere correlates with the amount of walking disability [18]. An optical imaging study shows a between-hemispheric imbalance of oxygenated hemoglobin level in the medial primary sensorimotor cortex that is greater in the unaffected hemisphere than in the affected hemisphere. A reduction of this asymmetry is associated with a favorable gait recovery [19]. A TMS study shows an interhemispheric asymmetry of corticomotor excitability of the legs to the detriment of the affected hemisphere, as well as a correlation between the reduction of this asymmetry and a favorable motor outcome [20]. Another TMS trial reveals increased connectivity between the contralesional hemisphere and the affected lower limb, which correlated with the amount of walking disability [18]. A diffusion-weighted MRI shows that the higher the anatomical connectivity between the ipsilesional M1 and the (a) cerebral peduncle, (b) thalamus, and (c) red nucleus, the better is the lower limb motor performance [21]. Furthermore, stroke-related disturbances of the spinal system were detected, as well as its relationship to gait disability. In fact, stroke patients show an increase of the H-reflex, in comparison to healthy subjects [22], and its normalization to be associated with a successful motor recovery of the walking ability [23].

### Non-invasive brain stimulation for network modulation

The application of non-invasive brain stimulation in rehabilitation aims at prolonged effects on the neural network. It is assumed that these techniques modulate synaptic connectivity, similar to long-term potentiation and long-term depression, which are considered relevant mechanism of plastic reorganization [24]. The amount and the duration of the induced neurophysiological changes depend on the stimulation intensity and duration [25, 26]. The available data indicates that a direct current of at least 0.6 mA that is applied for at least three minutes is sufficient to modulate cortical excitability beyond the stimulation period. Applying tDCS of 1 mA for five to seven minutes leads to short-term changes of cortical excitability that last 10–15 min after the end of stimulation. For long-term modulation of cortical excitability (1 h or more) a current of 1 mA need to be applied over a period of at least 11 min [25]. A single session of rTMS induces cortical excitability changes that last for at least 30 min after the end of stimulation [26]. In previous decades specific certain protocols have been established as either “facilitatory” or “inhibitory” for both rTMS and tDCS/tACS/tsDCS techniques. High-frequency rTMS (≥ 5 Hz), intermittent theta burst stimulation (iTBS), paired pulse rTMS (inter-stimulus interval 1.5 ms) and anodal tDCS/tACS are considered to have “up-regulating” effects on neural processing. In contrast low-frequency rTMS (1 Hz), continuous theta burst stimulation (cTBS), paired-pulse rTMS (inter-stimulus interval 3 ms) and cathodal tDCS are expected to induce a “down-regulation” [7, 8]. Indeed, several studies demonstrated a modulation of neural processing outside this framework [27–30]. An earlier study has shown that several rTMS protocols (1 Hz, 10 Hz, 15 Hz, 20 Hz) can induce an increase and a decrease of corticospinal excitability in the stimulated hemisphere [27]. Similarly, a more recent trial demonstrated inhibitory and facilitatory influences on corticospinal excitability of both iTBS and cTBS [28]. The TBS-induced effect was highly correlated with the pre-interventional MEP latency [28]. Similarly, cTBS decreased and increased corticospinal excitability, and its effects correlated with pre-interventional MEP variability and late I-wave recruitment [29]. Also, both anodal and cathodal tDCS induced increases and decreases of corticospinal excitability in the stimulated hemisphere, and the intervention-induced effects correlated with the pre-interventional MEP-latency [30]. Thus, present data shows that the responses to “up” and “down” regulating brain stimulation protocols are inconsistent, already in the healthy condition, and the factors influencing this inter-individual variability are not completely understood.

Another relevant issue is the state-dependency of stimulation effects. Specifically, brain state-dependent single-pulse TMS that was controlled by volitional modulation of sensorimotor beta-band oscillatory activity induced a robust increase of corticospinal excitability [31]. By contrast, the identical stimulation pattern applied independent of the brain state resulted in its decrease. The same was true, when single-pulse TMS was paired with peripheral stimulation; this pairing led to an increase or decrease of corticospinal excitability, when applied during volitional modulation of the sensorimotor beta-band activity or independent of the brain state, respectively. [32, 33].

There might be discrepancies, however, between online (i.e., during the intervention) and offline (i.e., after the intervention) stimulation effects, particularly, when the former is applied during behavioral tasks and the latter is done at rest. In this context, a meta-analysis detected timing- and cohort-dependent effects of anodal tDCS on the modulation of working memory. Healthy subjects demonstrated significant offline improvement but no online effects. By contrast, neuropsychiatric patients showed improved working memory during the stimulation but not afterwards [34]. Similarly, another meta-analysis detected timing-dependent effects of rTMS with regard to episodic memory, which was improved or deteriorated, when the stimulation was applied before or during the task [35]. Along the same lines a recent meta-analysis showed that rTMS and tDCS modulated visuospatial abilities in healthy subjects to a larger extent when applied before than during the task [36]. Therefore, we may assume that the effectiveness of non-invasive brain stimulation in supporting motor recovery after stroke will not depend on the stimulation protocol only, but also on the behavioral context.

### Non-invasive brain/spinal cord stimulation for improving walking, balance and lower limb motor function in stroke patients

Despite its limitations, the theory of interhemispheric imbalance and rivalry provides the most often used theoretical framework for the application of noninvasive brain stimulation in stroke rehabilitation [37]. Previous studies have demonstrated that several stroke-induced deficiencies, such as upper limb impairment or visuo-spatial disabilities may be successfully restored by application of non-invasive brain stimulation within this concept [38, 39]. The available data (see previous subchapter) indicates that a similar application may also be useful for supporting gait, balance, and lower limbs´ motor functioning. This means either “inhibitory” stimulation of the contralesional hemisphere or “facilitatory” stimulation of the ipsilesional hemisphere. Furthermore, it is conceivable that “inhibitory” spinal stimulation may be beneficial.

This systematic review and meta-analysis summarize the current evidence for non-invasive brain and spinal cord stimulation to support gait, balance and/or lower limbs function in stroke patients. The effectiveness is analyzed with regard to the technique used (rTMS, tDCS/tACS/tsDCS), protocols applied (anodal/cathodal/bilateral tDCS, low-frequency/high-frequency rTMS, iTBS/cTBS), stimulated hemisphere (affected/non-affected/ bilateral), stimulated area (primary motor cortex, cerebellum, supplementary motor area, spinal cord) and applied study design (stimulation amount, evaluation schedule). Furthermore, information regarding the participants (time since stroke, gender, stroke type and location), the exact stimulation location, and methodological quality of the trial is included.

## Methods

The protocol of this systematic review and meta-analysis bases on the Preferred Reporting Items for Systematic Reviews and Meta-Analysis (PRISMA) guidelines. A previous registration of the protocol was not performed.

### Search protocol

**Fig. 1:**
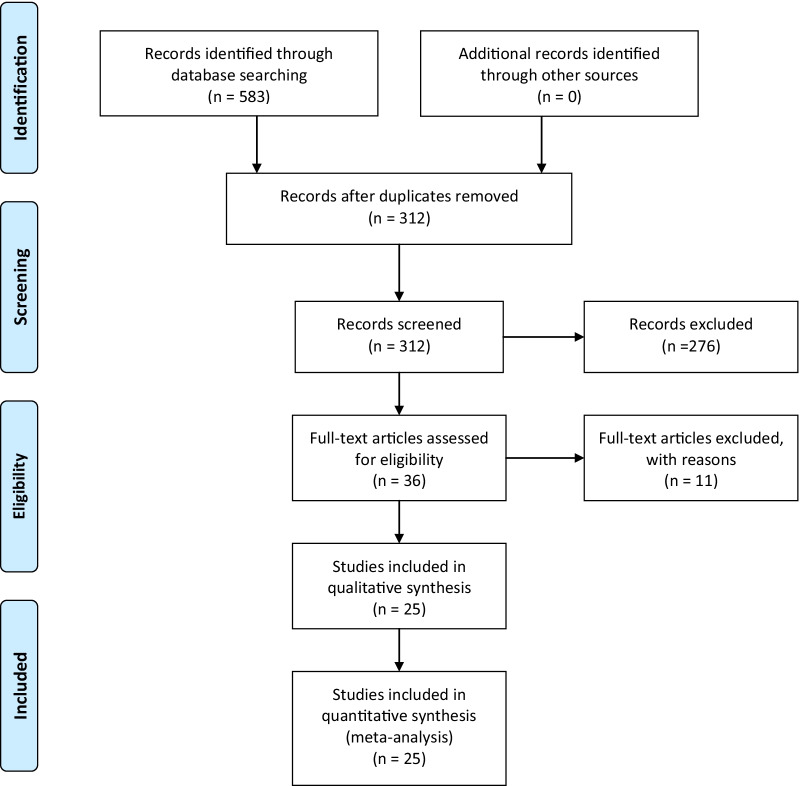
PRISMA flow diagram

Relevant studies were identified by searching of electronic database PubMed from inception to 31/03/2021. The combination of following search terms was used: (1) “tDCS” or “rTMS” or “tACS” or “tsDCS” and (2) “balance” or “postural control” or “gait” or “walking” or “lower limbs” and (3) stroke. The screening was performed by two independent reviewers (JV and AG). Disagreements were resolved by consensus. Figure 1 illustrates the search strategy based on the PRISMA guidelines.

### Eligibility criteria

Trials matching the following criteria were enrolled: (1) randomized controlled trials, (2) diagnosis stroke, (3) at least five participants per intervention, (4) rTMS, tDCS, tACS and/or tsDCS as intervention, (5) pre- and post-interventional assessments of gait, balance and/or lower limb motor function, (6) written in English or German.

### Data extraction

The data on gait, balance and/or lower limb function were extracted from the included publications. Depending on the availability, we considered either (1) the pre-intervention, post-intervention and follow up data, or (2) the difference between the pre-intervention and post-intervention data and the difference between the pre-intervention and follow up-data were obtained. The secondary data extracted were: (1) patients´ characteristics (number, gender, age, time since incident, stroke etiology and location), (2) stimulation parameters (technique used, positioning, duration, intensity, number of sessions), (3) methods (study design, assessments, evaluations schedule).

### Data synthesis

Based on the methodological approach, the included experiments were split up into (i) experiments comparing real brain stimulation with sham stimulation and (ii) experiments comparing two different brain stimulation protocols. Within this framework, a subcategorization was done depending on (a) stimulation technique (rTMS, tDCS/tACS/tsDCS), (b) stimulation positioning (affected hemisphere, non-affected hemisphere, bilateral, spinal) and (c) stimulation protocol (“facilitatory”: anodal tDCS/tACS/tsDCS, high-frequency rTMS, iTBS, “inhibitory”: cathodal tDCS/tACS/tsDCS, low-frequency rTMS, cTBS, and “combined”).

### Statistical analysis

Effect size calculators were used to estimate the effect size and the 95% confidence interval for each experiment [40, 41]. Depending on availability, the calculations are based on either means and standard deviations of pre-intervention, post-intervention and follow up data, or on means and standard deviations of the difference between the pre-intervention and post-intervention data and the difference between the pre-intervention and follow up-data. Where multiple assessments were applied, the effect sizes and the 95% confidence intervals were calculated for all outcomes, and on their basis average values were determinate for each experiment. The Cohen definition of effect size was used for interpretation (d ≥ 0.2 “small”, d ≥ 0.5 “medium”, d ≥ 0.8 “large”) [42]. The inconsistency test (I2) was applied to evaluate the homogeneity across experiments, where values above 50% indicate high heterogeneity [43].

### Methodological quality assessment

11-items PEDro scale was applied to evaluate the methodological quality of the studies, such as random allocation, subjects´ and assessors´ blinding, dropout rate etc [44]. The higher the total score, the higher the methodological quality (10–9 excellent, 8–6 good, 5–4 fair and < 4 poor).

## Results

### TDCS, tADCS, tsDCS

In total, 16 studies tested the effects of tDCS, tACS or tsDCS (Table 1) [45–60]. Their methodological quality varied between fair and excellent (Table 2).

**Table 1:** Overview of studies investigating tDCS/tACS/tsDCS in supporting gait, balance and/or lower limb motor function in stroke patients

References	Participants number, gender, age/time since stroke	Stroke etiology/location/affected hemisphere	Stimulation technique, intensity	Electrodes positioning		Study design/electrodes positioning technique	Number and duration of sessions/evaluations schedule	Outcomes
				Anode	Cathode			
Bornheim et al. [46]	31 males, 15 females/63 ± 12 years/2 ± 0 days	46 ischemic/na/26 right, 20 left	(1) 1.0 mA anodal tDCS	IL C3/C4	CL Fp2/Fp1	Parallel groups (23 + 23)/10–20 EEG system	20 sessions à 20 min/pre, post, two-week, three-month, six-month, and one-year follow-up	FMA-LE
			(2) sham tDCS					
Chang et al. [48]	15 males, 9 females/63 ± 11 years/16 ± 6 days	24 ischemic/na/13 right, 11 left	(1) 2.0 mA anodal tDCS	IL tibialis anterior M1	CL SO	Parallel groups (12 + 12)/TMS	10 sessions à 10 min/pre, post	FAC, BBS, MI-LE, FMA-LE, gait (cadence, speed, stride length, step length, step time)
			(2) sham tDCS					
Tahtis et al. [59]	11 males, 3 females/62 ± 12 years/23 ± 8 days	14 ischemic/8 subcortical, 6 cortical/8 right, 6 left	(1) 2.0 mA bilateral tDCS	IL 3.0 cm lateral to CZ	CL 3.0 cm lateral to CZ	Parallel groups (7 + 7)/10–20 EEG system	1 session à 15 min/pre, post	TUG, POMA
			(2) sham tDCS					
Manji et al. [53]	21males, 9 females/63 ± 11 years/33 ± 9 days	17 ischemic, 13 hemorrhagic/na /na	(1) 1.0 mA anodal + cathodal tDCS	3.5 cm anterior to CZ	inion	Crossover (30–30)/10–20 EEG system	2 × 5 session à 20 min/pre, post1, post2	10MWT, FMA-LE, TUG, TCT, POMA
			(2) sham tDCS					
Sayes et al. [57]	17 males, 14 females/63 ± 9 years/42 ± 18 days	26 ischemic, 5 hemorrhagic/7 subcortical, 24 cortical/14 right, 17 left	(1) 1.5 mA bilateral tDCS	IL C3/C4	CL C3/C4	Parallel groups (16–15)/10–20 EEG system	16 sessions à 20 min/pre, post	TT, RMI, TIS
			(2) sham tDCS					
Andrade et al. [45]	35 males, 25 females/69 ± 3 years/2.7 ± 0.5 months	na/na/na	(1) 2.0 mA anodal tDCS	IL C3/C4	CL SO	Parallel groups (15 + 15 + 15 + 15)/10–20 EEG system	10 sessions à 20 min/pre, post, one-month, and three-month follow-up	FSST, OSI; BBS, FES-I, STS, 6MWT,
			(2) 2.0 mA cathodal tDCS	IL SO	CL C3/C4			
			(3) 2.0 mA bilateral tDCS	IL C3/C4	CL C3/C4			
			(4) sham tDCS					
Klomjai et al. [50]	14 males, 5 females/57 ± 3 years/3.2 ± 1,7 months	19 ischemic/16 subcortical, 3 cortical/12 right, 7 left	(1) 2.0 mA bilateral tDCS	IL M1	CL M1	Crossover (19–19)/10–20 EEG system	2 × 1 session à 20 min/pre1, post1, pre2, post2	TUG, STS, MVC (knee extensor)
			(2) sham tDCS					
Geroin et al. [49]	14 males, 6 females/63 ± 7 years/26 ± 6 months	na/15 cortical, 5 subcortical,/na	(1) 1.5 mA anodal tDCS	IL leg M1	CL SO	Parallel groups (10 + 10)/na	10 sessions à 7 min/pre, post, two-weak follow-up	6MWT, 10MWT, FAC, MI-LE, RMI, gait (cadence, symmetry, support time)
			(2) sham tDCS					
Picelli et al. [56]	22 males, 8 females/63 ± 8 years/4.7 ± 2.8 years	30 ischemic/11 subcortical, 19 cortical/na	(1) 2.0 mA anodal tDCS + sham tsDCS	IL C3/C4	CL SO	Parallel groups (10 + 10 + 10)/10–20 EEG system	10 sessions à 40 min/pre, post, two-week, and four-week follow-up	6MWT, gait (cadence, support time)
			(2) 2.5 mA cathodal tsDCS + sham tDCS	NA shoulder	10Th			
			(3) 2,0 mA anodal tDCS + 2.5 mA cathodal tsDCS	IL C3/C4 + NA shoulder	CL S0 + 10Th			
Picelli et al. [55]	13 males, 7 females/63 ± 12 years/5.0 ± 3.7 years	20 ischemic/7 subcortical, 13 cortical/na	(1) 2.0 mA cathodal tDCS	A bucinator muscle	CL O1/O2	Parallel groups (10 + 10)/10–20 EEG system	10 sessions à 20 min/pre, post, two-week, and four-week follow-up	6MWT, FAC, MI-LE, MAS, gait (cadence, support time)
			(2) 2.0 mA anodal tDCS	IL leg M1	CL SO			
Picelli et al. [54]	21 males, 19 females/65 ± 10 years/5.3 ± 3.7 years	40 ischemic, 14 subcortical, 26 cortical/na	(1) 2.0 mA cathodal tDCS	A bucinator muscle	CL O1/O2	Parallel groups (20 + 20)/10–20 EEG system	10 sessions à 20 min/pre, post, two two-week, and four-week follow-up	6MWT, gait (cadence, support time)
			(1) 2.0 mA cathodal tDCS	NA bucinator muscle	IL O1/O2			
Madhavan et al. [52]	30 males, 10 females/60 ± 9 years/5.8 ± 4.6 years	25 ischemic, 14 hemorrhagic, 1 na/na/na	(1) 2.0 mA anodal tDCS	IL leg M1	CL SO	Parallel groups (20 + 20)/10–20 EEG system	12 sessions à 15 min/pre, post, three months follow-up	10MWT, 6MWT, BBS, TUG, mini-BEST, FMA-LE, ABC
			(2) sham tDCS					
Koganemaru et al. [51]	8 males, 3 females/66 ± 4 years/6.2 ± 2.6 years	4 ischemic/7 hemorrhagic/11 subcortical/na	(1) 0.0–2.0 mA bilateral tACS	IL tibialis anterior M1	CL 3.0 cm lateral and 3.0 cm rostral to inion	Crossover (11–11)/TMS	2 × 1 session à 20 min/pre1, post1, pre2, post2	10MWT, VAS, MAS
			(2) sham tACS			Crossover (8–8)/TMS	2 × 5 sessions à 20 min/pre1, post1, one-week follow-up1, pre2, post2, one-week follow-up2	10MWT, 6MWT, TUG, gait (gait speed, joint angles), mini-BEST, VAS, MAS
Zandvliet et al. [60]	12 males, 3 females/57 ± 10 years/9 ± 12 years	11 ischemic, 4 hemorrhagic/2 subcortical, 13 cortical/9 right, 6 left	(1) 1.5 mA anodal tDCS	IL 3.0 cm lateral to inion	NA bucinator muscle	Crossover (15–15-15)/10–20 EEG system	3 × 1 session à 20 min/pre1, post1, pre2, post2, pre3, post3	eyes open, eyes closed and tandem stance (center of pressure amplitude, velocity, range)
			(2) 1.5 mA anodal tDCS	CL 3.0 cm lateral to inion	A bucinator muscle			
			(3) sham tDCS					
Seo et al. [58]	16 males, 5 females/62 ± 9 years/9.5 ± 8.6 years	16 ischemic, 5 hemorrhagic/na/13 right, 8 left	(1) 2.0 mA anodal tDCS	IL leg M1	CL SO	Parallel groups (11 + 10)/10–20 EEG system	10 sessions à 20 min/pre, post, four-week follow-up	10MWT, 6MWT, FAC, BBS, FMA-LE, MRC (hip, knee, ankle)
			(2) sham tDCS					
Cattagni et al. [47]	19 males, 5 females/57 ± 13 years/10 ± 7 years	17 ischemic, 7 hemorrhagic/na/13 right, 11 left	(1) 2.0 mA anodal tDCS	IL leg M1	CL SO	Crossover (24–24)/10–20 EEG system	2 × 1 session à 30 min/pre, post	gait (speed, step length)
			(2) sham tDCS					
Picelli et al. [55]	13 males, 7 females/63 ± 12 years/5.0 ± 3.7 years	20 ischemic/7 subcortical, 13 cortical/na	(1) 2.0 mA cathodal tDCS	A bucinator muscle	CL O1/O2	Parallel groups (10 + 10)/10–20 EEG system	10 sessions à 20 min/pre, post, two-week, and four-week follow-up	6MWT, FAC, MI-LE, MAS, gait (cadence, support time)
			(2) 2.0 mA anodal tDCS	IL leg M1	CL SO			
Picelli et al. [54]	21 males, 19 females/65 ± 10 years/5.3 ± 3.7 years	40 ischemic, 14 subcortical, 26 cortical/na	(1) 2.0 mA cathodal tDCS	A bucinator muscle	CL O1/O2	Parallel groups (20 + 20)/10–20 EEG system	10 sessions à 20 min/pre, post, two two-week, and four-week follow-up	6MWT, gait (cadence, support time)
			(1) 2.0 mA cathodal tDCS	NA bucinator muscle	IL O1/O2			
Madhavan et al. [52]	30 males, 10 females/60 ± 9 years/5.8 ± 4.6 years	25 ischemic, 14 hemorrhagic, 1 na/na/na	(1) 2.0 mA anodal tDCS	IL leg M1	CL SO	Parallel groups (20 + 20)/10–20 EEG system	12 sessions à 15 min/pre, post, three months follow-up	10MWT, 6MWT, BBS, TUG, mini-BEST, FMA-LE, ABC
			(2) sham tDCS					
Koganemaru et al. [51]	8 males, 3 females/66 ± 4 years/6.2 ± 2.6 years	4 ischemic/7 hemorrhagic/11 subcortical/na	(1) 0.0–2.0 mA bilateral tACS	IL tibialis anterior M1	CL 3.0 cm lateral and 3.0 cm rostral to inion	Crossover (11–11)/TMS	2 × 1 session à 20 min/pre1, post1, pre2, post2	10MWT, VAS, MAS
			(2) sham tACS			Crossover (8–8)/TMS	2 × 5 sessions à 20 min/pre1, post1, one-week follow-up1, pre2, post2, one-week follow-up2	10MWT, 6MWT, TUG, gait (gait speed, joint angles), mini-BEST, VAS, MAS
Zandvliet et al. [60]	12 males, 3 females/57 ± 10 years/9 ± 12 years	11 ischemic, 4 hemorrhagic/2 subcortical, 13 cortical/9 right, 6 left	(1) 1.5 mA anodal tDCS	IL 3.0 cm lateral to inion	NA bucinator muscle	Crossover (15–15-15)/10–20 EEG system	3 × 1 session à 20 min/pre1, post1, pre2, post2, pre3, post3	eyes open, eyes closed and tandem stance (center of pressure amplitude, velocity, range)
			(2) 1.5 mA anodal tDCS	CL 3.0 cm lateral to inion	A bucinator muscle			
			(3) sham tDCS					
Seo et al. [58]	16 males, 5 females/62 ± 9 years/9.5 ± 8.6 years	16 ischemic, 5 hemorrhagic/na/13 right, 8 left	(1) 2.0 mA anodal tDCS	IL leg M1	CL SO	Parallel groups (11 + 10)/10–20 EEG system	10 sessions à 20 min/pre, post, four-week follow-up	10MWT, 6MWT, FAC, BBS, FMA-LE, MRC (hip, knee, ankle)
			(2) sham tDCS					
Cattagni et al. [47]	19 males, 5 females/57 ± 13 years/10 ± 7 years	17 ischemic, 7 hemorrhagic/na/13 right, 11 left	(1) 2.0 mA anodal tDCS	IL leg M1	CL SO	Crossover (24–24)/10–20 EEG system	2 × 1 session à 30 min/pre, post	gait (speed, step length)
			(2) sham tDCS					

*BBS*   Berg Balance Scale, *CL*   contralesional, *cm*   centimeter, *EEG*   electroencephalography, *FAC*   Functional Ambulation Categories, *FES-I*   Falls Efficacy Scale—International, *FMA-LE*   Fugl-Meyer Assessment—Lower Extremity, *FSST*   Four Square Step Test, *IL*   ipsilesional, *mA*   milliampere, *min*   minute, *MI-LE*   Motoricity Index—Lower Extremity, *MRI*   magnetic resonance imaging, *MVC*   maximal voluntary contraction, *M1*   primary motor cortex, *na*   not available/not applicable, *NA*   non-affected, *OSI*   Overall Stability Index, *POMA*   Performance Oriented Mobility Assessment, *RMI*   Rivermead Mobility Index, *SO*   supra-orbital, *STS*   Sit to Stand Test, *tADS*   transcranial alternating current stimulation, *TCT* Trunk Control Test, *tDCS*   transcranial direct current stimulation, Th10   the tenth thoracic vertebra, *TIS*   Trunk Impairment Scale, *TMS*   transcranial magnetic stimulation, *tsDCS*   trans-spinal direct current stimulation, *TT*   Tinetti Test, *TUG*   Timed Up and Go Test, *6MWT*   Six-Minute Walk Test, *10MWT*   10-Meter Walking Test, *A*   affected, *ABC*   Activities-Specific Balance Confidence Scale, *BBS*   Berg Balance Scale, *CL*   contralesional, *cm*   centimeter, *EEG*   electroencephalography, *FAC*   Functional Ambulation Categories, *FMA-LE*   Fugl-Meyer Assessment—Lower Extremity, *mA*   milliampere, *MAS*   Modified Ashworth Scale, *min*   minute, *MI-LE*   Motoricity Index—Lower Extremity, *IL*   ipsilesional, *min*   minute, *mini-BEST*   mini-Balance-Evaluation-System-Test, *MRC*   Medical Research Council, *M1*   primary motor cortex, *NA*   non-affected, *SO*   supra-orbital, *VAS*   Visual Analog Scale,

**Table 2:** Methodological quality of the included studies—assessed with the 11-item PEDro scale

PEDro scale items	tDCS/tACS/tsDCS studies																	rTMS studies								
	Bornheim et al. [46]	Chang et al. [48]	Tahtis et al. [59]	Manji et al. [53]	Sayes et al. [57]	Andrade et al. [45]	Klomjai et al. [50]	Geroin et al. [49]	Madhavan et al. [52]	Picelli et al. [56]	Picelli et al. [54]	Picelli et al. [55]	Madhavan et al. [52]	Koganemaru et al. [51]	Zandvliet et al. [60]	Seo et al. [58]	Cattagni et al. [47]	Sasaki et al. [67]	Kim et al. [63]	Huang et al. [62]	Lin et al. [65]	Koch et al. [64]	Chiefflo et al. [61]	Wang et al. [20]	Rastgoo et al. [66]	Wang et al. [68]
Eligibility criteria specified	+	+	+	+	+	+	+	+	+	+	+	+	+	+	+	+	+	+	+	+	+	+	+	+	+	+
Random allocation	1	1	1	1	1	1	1	1	1	1	1	1	1	0	1	1	1	1	1	1	1	1	1	1	1	1
Concealed allocation	1	0	0	0	1	0	0	1	0	1	1	1	1	0	0	1	0	0	1	0	0	1	1	1	0	0
Comparable baseline	1	1	1	1	1	1	1	1	1	1	1	1	1	1	1	1	1	1	1	1	1	1	1	1	1	1
Subject blinding	1	1	1	1	1	1	1	1	1	1	1	1	1	1	1	1	1	1	1	1	1	1	1	1	1	1
Therapist blinding	1	0	0	1	0	0	0	0	0	0	0	0	0	0	0	1	0	1	0	1	1	0	1	0	0	0
Assessor blinding	1	1	1	0	1	1	1	1	0	1	1	1	1	0	0	1	1	1	1	1	0	1	1	1	0	1
Less than 15% dropouts	1	1	1	1	1	1	1	1	1	1	1	1	1	1	1	1	1	1	1	1	1	1	1	1	1	1
Intention-to-treat analysis	1	0	1	1	1	1	0	1	0	1	1	1	1	0	1	0	0	1	1	0	1	0	0	0	0	0
Between-group comparison	1	1	1	1	1	1	1	1	1	1	1	1	1	1	1	1	1	1	1	1	1	1	1	1	1	1
Point estimates and variability	1	1	1	1	1	1	1	1	1	1	1	1	1	1	1	1	1	1	1	1	1	1	1	1	1	1
PEDro score total (0–10)	##	7	8	8	9	8	7	9	6	9	9	9	9	5	7	9	7	9	9	8	8	8	9	8	6	7

*PEDro*   Physiotherapy Evidence Database, *rTMS*   repetitive transcranial magnetic stimulation, *tACS*   transcranial alternating current stimulation, *tDCS*   transcranial dirrect current stimulation, *tsDCS*   trans-spinal dirrect current stimulation

*Participants:* Overall 445 stroke patients (299 males, 146 females) were investigated. The cohorts were inconsistent in term of the mean time since stroke (between two days and ten years), stroke etiology (309 ischemic, 55 hemorrhagic, 81 na), lesion location (91 subcortical, 109 cortical, 245 na) and lesion site (108 right, 86 left, 251 na).

*Study design:* 14 studies investigated the effectiveness of real tDCS/tACS/tsDCS in comparison to sham stimulation [45–53, 56–60]. Five studies compared different stimulation protocols [45, 54–56, 60]. The overall duration of active stimulation varied between 15 and 400 min (one to 16 sessions with a duration of seven to 40 min were applied). All studies performed a pre- and post-evaluation of the parameters assessed. Nine studies performed additional follow-up evaluations, one up to 24 weeks after completing the intervention [45, 46, 49, 51, 52, 54–56, 58].

*Stimulation protocol:* Seven studies applied anodal tDCS over the ipsilesional hemisphere [45, 47, 49, 52, 58, 60]. Five trials investigated bilateral tDCS (combining anodal tDCS over the ipsilesional hemisphere and cathodal tDCS over the contralesional hemisphere) [45, 50, 57, 59] or bilateral tACS [51]. The contralesional hemisphere alone was stimulated in only two studies – in one study with anodal [60] and in another with cathodal tDCS [45]. One study combined anodal tDCS over supplementary motor area with cathodal tDCS over cerebellum [53]. One trial investigated cathodal tsDCS [56].

**Fig. 2:**
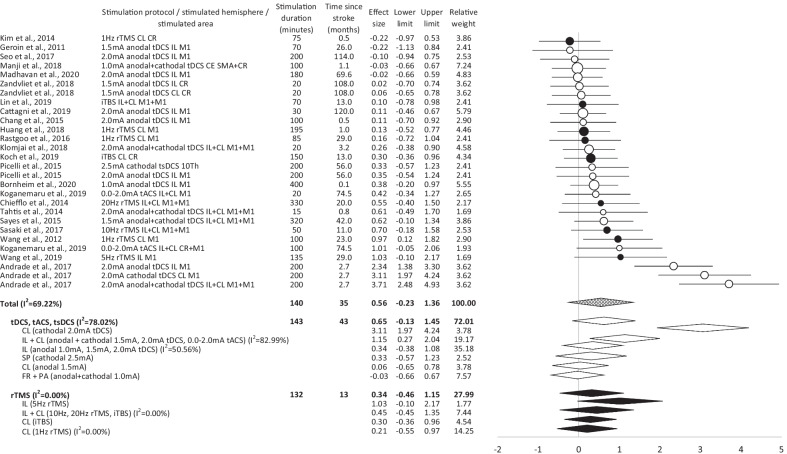
Postinterventional overview of effect sizes, 95% confidence intervals and homogeneity for studies comparing non-invasive brain stimulation/spinal cord stimulation with sham stimulation for improving gait, balance and/or lower limb recovery in stroke patients. *black*   repetitive transcranial magnetic stimulation, *white*   transcranial/trans-spinal direct current/alternating current stimulation, *patterned*   non-invasive brain stimulation overall, *CL*   contralesional, *FR*   frontal, *Hz*   hertz, *IL*   ipsilesional, *I*^*2*^   inconsistency test, *iTBS*   intermittent theta burst stimulation, *mA*   milliampere, *PA*   parietal, *rTMS*   repetitive transcranial magnetic stimulation, *SP*   spinal, *tACS*   transcranial alternating current stimulation, *tDCS*   transcranial direct current stimulation, *tsDCS*   trans-spinal direct current stimulation

*Effectiveness—active stimulation versus sham:* In total, the post-interventional data show a medium-sized effect of tDCS/tACS/tsDCS on gait, balance, and lower limb motor function in stroke patients (Fig. 2). However, effects were inhomogeneous across the studies. Cathodal tDCS over the contralesional hemisphere and bilateral tDCS induce large effects on the evaluated parameters. In contrast, only small effects were found for anodal tDCS/tACS over the ipsilesional hemisphere and for tsDCS. No effect was induced by anodal tDCS over the contralesional hemisphere and by fronto-parietal tDCS.

**Fig. 3:**
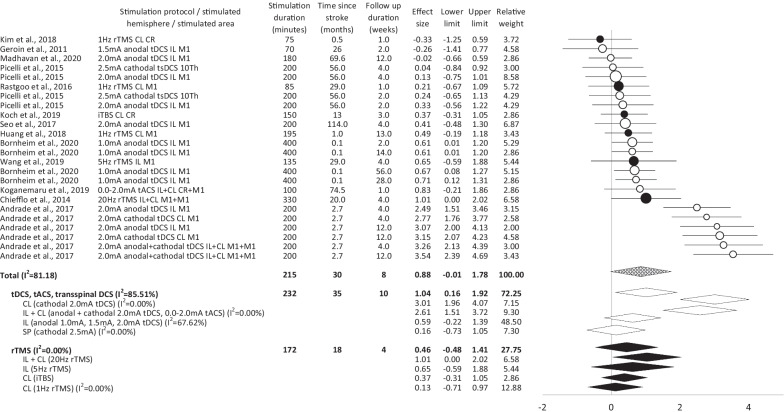
Follow-up overview of effect sizes, 95% confidence intervals and homogeneity for studies comparing non-invasive brain stimulation/spinal cord stimulation with placebo stimulation in supporting gait, balance and/or lower limb recovery in stroke patients. *black*   repetitive transcranial magnetic stimulation, *white*   transcranial/trans-spinal direct current/alternating current stimulation, *patterned*   non-invasive brain stimulation overall, *CL*   contralesional, *Hz*   hertz, *IL*   ipsilesional, *I*^*2*^   inconsistency test, *iTBS*   intermittent theta burst stimulation, *mA*   milliampere, *rTMS*   repetitive transcranial magnetic stimulation, *SP*   spinal, *tACS*   transcranial alternating current stimulation, *tDCS*   transcranial direct current stimulation, *tsDCS*   trans-spinal direct current stimulation

The follow-up data demonstrate a large-sized effect of stimulation on the assessed parameters, as well as inhomogeneity of the effects across the experiments (Fig. 3). Cathodal tDCS over the contralesional hemisphere and bilateral tDCS evoke large effects. A medium-sized effect was detected for anodal tDCS/tACS over the ipsilesional hemisphere. No effect was found for tsDCS.

**Fig. 4:**
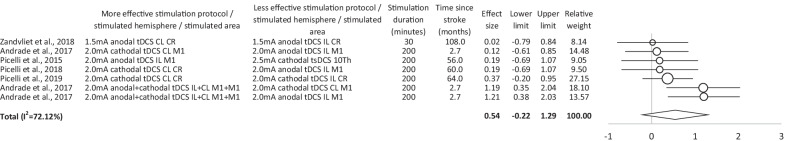
Postinterventional overview of effect sizes, 95% confidence intervals and homogeneity for studies comparing different non-invasive brain stimulation protocols in supporting gait, balance and/or lower limb recovery in stroke patients. *white*   transcranial/trans-spinal direct current stimulation, *CL*   contralesional, *IL*   ipsilesional, *I*^*2*^   inconsistency test, *mA*   milliampere, *tDCS*   transcranial direct current stimulation, *tsDCS*   trans-spinal direct current stimulation

**Fig. 5:**
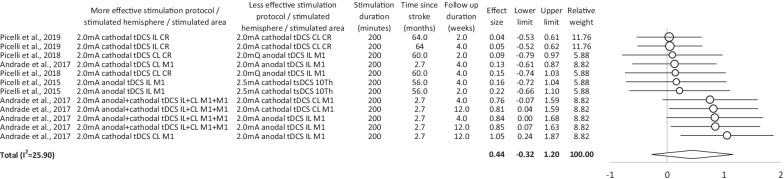
Follow-up overview of effect sizes, 95% confidence intervals and homogeneity for studies comparing different non-invasive brain stimulation protocols in supporting gait, balance and/or lower limb recovery in stroke patients. *white*   transcranial/trans-spinal direct current stimulation, *CL*   contralesional, *IL*   ipsilesional, *I*^*2*^   inconsistency test, *mA*   milliampere, *tDCS*   transcranial direct current stimulation, *tsDCS*   trans-spinal direct current stimulation

*Effectiveness—comparison of different stimulation protocols:* Both the post-interventional and the follow-up data demonstrate that different stimulation protocols may induce significantly different effects on gait, balance, and lower limbs motor function in stroke patients (Figs. 4, 5). In particular bilateral tDCS/tACS and cathodal tDCS over the contralesional hemisphere are superior to other stimulation protocols.

### rTMS

Nine studies tested rTMS for improving gait, balance, and lower limb motor function in stroke patients (Table 3) [20, 61–68]. Their methodological quality was good to excellent (Table 2).

**Table 3:** Overview of studies investigating rTMS in supporting gait, balance and/or lower limb motor function in stroke patients

References	Participants number, gender, age/time since stroke	Stroke etiology/location/affected hemisphere	Stimulation parameters, stimulated area	Study design/rTMS positioning technique	Number and duration of sessions/evaluations schedule	Outcomes
Sasaki et al. [67]	13 males, 8 females/11 ± 7 days	11 ischemic, 10 hemorrhagic/na/9 right, 12 left	(1) 10 Hz bilateral rTMS (1000 pulses, 90% rMT, double-cone coil) over leg M1	Parallel groups (11 + 10)/TMS	5 sessions à 10 min/pre, post	BRS
			(2) sham rTMS (sham coil)			
Kim et al. [63]	17 males, 15 females/16 ± 9 days	32 ischemic/9 subcortical, 23 brain stem/na	(1) 1 Hz unilateral rTMS (900 pulses, 100% rMT, figure-of-eight coil) over contralesional cerebellum	Parallel groups (22 + 10)/10–20 EEG system	5 sessions à 15 min/pre, post, one month follow up	10MWT, BBS
			(2) sham rTMS (perpendicular coil positioning)			
Huang et al. [62]	23 males, 15 females/29 ± 22 days	25 ischemic, 13 hemorrhagic/28 subcortical, 10 cortical/17 right, 21 left	(1) 1 Hz unilateral rTMS (900 pulses, 120% rMT, double-cone coil) over contralesional musculus rectus femoris M1	Parallel groups (18 + 20)/TMS	13 sessions à 15 min/pre, post, three months follow up	TUG, PASS, FMA-LE
			(2) sham rTMS (sham coil)			
Lin et al. [65]	17 males, 3 females/13 ± 7 months	16 ischemic, 4 hemorrhagic/9 right, 11 left	(1) iTBS bilateral (1200 pulses, 100% rMT, figure-of-eight coil) over musculus rectus femoris M1	Parallel groups (10 + 10)/TMS	10 sessions à 7 min/pre, post	BBS, TUG, 10MWT, FMA-LE
			(2) sham rTMS (sham coil)			
Koch et al. [64]	21 males, 13 females/13 ± 17 months	34 ischemic/17 subcortical, 17 cortical/20 right, 14 left	(1) iTBS unilateral (1200 pulses, 80% aMT, figure-of-eight coil) over contralesional lateral cerebellum	Parallel groups (17 + 17)/neuronavigation	15 sessions à 10 min/pre, post, three weeks follow up	BBS
			(2) sham iTBS (na)			
Chiefflo et al. [61]	na/61 ± 10 years/20 ± 7 months	5 ischemic, 4 hemorrhagic/9 subcortical/5 right, 4 left	(1) 20 Hz bilateral rTMS (1500 pulses, 90% rMT, H12-coil) over leg M1	Crossover (9–9)/TMS	2 × 11 sessions à 30 min/pre1, post1, four weeks follow up1, pre2, post2, four weeks follow up2	FMA-LE, 10MWT, 6MWT
			(2) sham rTMS (sham coil)			
Wang et al. [20]	15 males, 9 females/23 ± 14 months	na/na/14 right, 10 left	(1) 1 Hz rTMS unilateral (600 pulses, 90% rMT, figure-of-eight coil) over contralesional musculus rectus femoris M1	Parallel groups (14 + 14)/TMS	10 sessions à 10 min/pre, post	FMA-LE, gait analysis (speed, cadence, step length, support time, asymmetry)
			(2) sham rTMS (perpendicular coil positioning)			
Rastgoo et al. [66]	16 males, 4 females/29 ± 19 months	15 ischemic, 5 hemorrhagic/15 subcortical, 5 cortical/13 right, 7 left	(1) 1 Hz rTMS unilateral (1000 pulses, 90% rMT, figure-of-eight coil) over contralesional leg M1	Crossover (10–10)/TMS	2 × 5 sessions à 17 min/pre1, post1, one weeks follow up1, pre2, post2, one weeks follow up2	TUG, FMA-LE
			(2) sham rTMS (sham coil)			
Wang et al. [68]	11 males, 3 females/29 ± 20 months	6 ischemic, 8 hemorrhagic/na/8 right, 6 left	(1) 5 Hz rTMS unilateral (900 pulses, 90% rMT, figure-of-eight coil) over ipsilesional musculus tibialis anterior M1	Parallel groups (8 + 6)/TMS	9 sessions à 15 min/pre, post, one month follow up	FMA-LE, gait analysis (speed, asymmetry)
			(2) sham rTMS (perpendicular coil positioning)			

*BBS*   Berg Balance Scale, *BRS*   Brunnstrom Recovery Stages, *EEG*   electroencephalography, *FMA-LE*   Fugl-Meyer Assessment—Lower Extremity, *Hz*   hertz, *iTBS*   intermittent theta burst stimulation, *M1*   primary motor cortex, *min*   minute, *na*   not applicable/not available, *PASS*   Postural Assessment Scale for Stroke Patients, *rMT*   resting motor threshold, *(r)TMS*   (repetitive) transcranial magnetic stimulation, *TUG*   Timed Up and Go Test, *6MWT*   Six-Minute Walk Test, *10MWT*   10-Meter Walking Test

*Participants:* In total, 212 patients (133 males, 70 females, 9 gender na) were enrolled. The study cohorts were inconsistent regarding the mean time since stroke (11 days to 29 months), stroke etiology (144 ischemic, 44 hemorrhagic, 24 na), stroke location (78 subcortical, 32 cortical, 23 brainstem, 79 na) and lesioned site (95 right, 85 left, 32 na).

*Study design:* All studies investigated the effectiveness of rTMS in comparison to sham stimulation. A direct comparison of different stimulation protocols was not performed. The duration of the total amount of active stimulation varied between 15 and 330 min (between five and 13 sessions with a duration of seven to 30 min were performed). All trials applied pre- and post-interventional assessments. Additional follow-up evaluations, over one week to three months after completing the stimulation, were performed in six trials [61–64, 66, 68].

*Stimulation protocol:* Four studies applied low-frequency rTMS over the contralesional hemisphere [62, 63, 66, 68]. Three trials performed bilateral stimulation (combining high-frequency rTMS or iTBS over the ipsilesional and the contralesional hemisphere) [61, 65, 67]. One study tested high-frequency rTMS over the ipsilesional hemisphere [68]. One trial applied iTBS over the contralesional hemisphere [64].

*Effectiveness—active stimulation versus sham:* In total, the post-interventional data indicate a small effect of rTMS on the observed parameters (Fig. 2). Despite a high homogeneity of detected effects, stimulation protocol dependent differences were found. High-frequency rTMS over the ipsilesional hemisphere induces large effects. ITBS over the contralesional hemisphere and bilateral stimulation evoke a small effect. No effect was found for low-frequency rTMS over the contralesional hemisphere.

The follow-up data show (in total) a middle-sized effect of rTMS on gait, balance, and lower limb motor function in stroke patients (Fig. 3). Despite the high homogeneity of effect sizes, stimulation protocol dependent differences were observed. Bilateral stimulation induces large effects. High-frequency rTMS over the ipsilesional hemisphere evokes middle-sized effects. ITBS over contralesional hemisphere results in a small effect. No effect is induced by low-frequency rTMS over the contralesional hemisphere.

## Discussion

Our data demonstrates that non-invasive neuromodulation is an effective way for improving gait, balance and/or lower limb motor function in stroke patients. This observation is supported by previous reviews and meta-analyses [69–72] that also demonstrate outcome-dependent effects [71, 72]. A recent meta-analysis revealed for example, significant effects of tDCS on functional ambulation category, Rivermead Mobility, and timed up and go test, but not on walking speed, 6-min walking distance, Tinetti test and Berg Balance Scale [71]. Similarly, another meta-analysis detected no relevant effects of rTMS on the Berg Balance Scale, while the remaining outcome measures for gait, balance and lower limb motor function were significantly influenced by the treatment [72]. In contrast to these studies, our meta-analysis focuses on the effectiveness in relation to the stimulation technique, protocol, hemisphere, area, duration and time since stroke (Table 4).

**Table 4:** Cross-tabulation of postinterventional effect sizes for studies evaluating non-invasive brain stimulation for improving gait, balance and/or lower limb recovery in stroke patients

	Stimulation induced effects on gait, balance and/or lower limb recovery (number of studies)									
	tDCS/tACS					rTMS				
	Small negative effect	No effect	Small positive effect	Medium positive effect	Large positive effect	Small negative effect	No effect	Small positive effect	Medium positive effect	Large positive effect
Overall										
	1	7	5	2	4	1	3	1	2	2
*Stimulated hemisphere*										
Affected	1	5	2		1					1
Non-affected		1			1	1	2	1		1
Biateral			2	2	2			1	2	
Centred		1								
Spinal			1							
*Stimulated areas*										
M1	1	4	3		2		2			2
CR		2				1		1		
M1 + M1			1	2	1		1		2	
M1 + CR					1					
SMA + M1		1								
10Th			1							
*Stimulation protocol*										
1 Hz rTMS						1	2			1
5 Hz rTMS										1
10 Hz rTMS									1	
20 Hz rTMS									1	
iTBS								1		
0.0–2.0 mA tACS			1		1					
1.0 mA anodal tDCS			1							
1.5 mA anodal tDCS	1	2								
2.0 mA anodal tDCS		4	1		1					
1.0 mA anodal + cathodal tDCS		1								
1.5 mA anodal + cathodal tDCS										
2.0 mA anodal + cathodal tDCS			1	1	1					
2.0 mA cathodal tDCS					1					
2.5 mA cathodal tsDCS			1							
*Stimulation duration*										
≥ 99 min	1	3	2	1		1	2		1	
100–199 min		3			1		1	1		2
200–299 min		1	2		3					
≤ 300 min			1	1					1	
*Time since stroke*										
0.1–3.2 months		2	2	1	3	1	1			
11.0–29.0 months	1		0				2	1	2	2
42.0–120.0 months		5	3	1	1					

No effect (d < 0.2), small effect (d = 0.2–0.49), medium effect (d = 0.5–0.79), large effect (d ≥ 0.8), *CR*   cerebellum, *Hz*   hertz, *iTBS*   intermittent theta burst stimulation, *mA*   milliampere, *M1*   primary motor cortex, *rTMS*   repetitive transcranial magnetic stimulation, *SMA*   supplementary motor area, *tACS*   transcranial alternating current stimulation, *tDCS*   transcranial direct current stimulation, *tsDCS*   trans-spinal direct current stimulation, *10Th*   the tenth thoracic vertebra

### Stimulation technique-dependent effects

Both, the post-interventional data, and the follow-up data indicate that tDCS/tACS/tsDCS is superior to rTMS in supporting gait, balance, and lower limb function in stroke patients. These findings contrast with observations made in previous meta-analyses, that indicate superior effects of rTMS (in comparison to tDCS) on balance and postural control [70], or on hemi-spatial neglect [36] in this cohort. Furthermore, our data detects heterogeneity of tDCS/tACS/tsDCS effects that did not occur in rTMS trials. Similar observations were not made previously [69, 70]. It is an open question whether and to what extent the detected differences reflect the differential impact of various techniques on the central nervous system and behavior, and which role other factors (such as patients characteristics, study design, and “outlier” etc.) may play. Our data shows that the superior effect of tDCS/tACS/tsDCS is primarily based on very high effects detected in a single study, which performed three different experiments in large cohorts [45]. Therefore, the results of this study strongly influence the overall outcome of the meta-analysis. The reason for the superior stimulation effectiveness in this specific study may be the high amount of therapy applied in an early phase after the incident (a more detailed discussion of this topic is included below). From a global perspective, our data reveals that available rTMS and tDCS/tACS/tsDCS trials differ significantly with respect to the time since stroke, amount of intervention, scheduling of the evaluations and study design (Figs. 2, 3, 4). While an average of 143 min (and 232 min in follow up data) of active treatment was applied in TDCS/tACS/tsDCS trials, an average of only 132 min (and 172 min in follow up data) was administrated in rTMS studies. This discrepancy may explain lower effect sized induced by rTMS interventions. The present data indicates that the behavioral changes are crucially determined by the amount of stimulation applied. Studies with relevant effects (according to Cohen’s effect size definition) applied on average 169 min (and 232 min on follow up) of stimulation, while studies without relevant effects applied 99 min (and 172 min on follow up) of stimulation only. Furthermore, the data shows stimulation technique-dependent differences that indicate that only a higher amount of tDCS/tACS/tsDSC but not of rTMS induces larger behavioral changes. tDCS/tACS/tsDSC studies with statistically relevant effects applied on average 186 min (and 241 min on follow up) of stimulation. tDCS/tACS/tsDSC trials without relevant effects applied 89 min (and 196 min on follow up) of stimulation only. By contrast, rTMS was applied for 132 min (172 min on follow up) and 134 min (no follow up data) in studies with and without relevant effects, respectively. Our observations do not confirm a previous meta-analysis that suggested that a larger number of rTMS sessions corresponded to more benefits for balance and postural control in stroke patients [70]. Furthermore, significantly different stroke chronicity was detected in rTMS trials as compared with tDCS/tACS/tsDCS studies. While on average 43 months (and 35 months in follow up data) passed by since stroke in tDCS/tACS/tsDCS trials, there were only 13 months (18 months in follow up data) since the incident in rTMS studies. This is surprising, since the available data indicates that the chronicity of stroke correlates with less motor recovery, and reduced therapy-induced benefits. A constraint-induced movement therapy applied over two weeks, for example, induces a greater improvement of motor function of the affected hand in patients who are less than 9 months post-stroke than in patients who are more than 12 months post-stroke [73]. Moreover, the follow-up duration is on average ten weeks in tDCS/tACS/tsDCS studies and only four weeks in rTMS trials. Furthermore, no rTMS trial performs a direct comparison of different protocols, in contrast to tDCS/tACS/tsDCS studies.

### Stimulation protocol-dependent effects

Both the post-interventional and the follow-up data demonstrate that different stimulation protocols induce differential effects on gait, balance, and lower limb motor function in stroke patients. In sum, bilateral stimulation is superior to unilateral protocols. Similarly, a previous meta-analysis demonstrates the superior effectiveness of bilateral tDCS/rTMS for improving visuo-spatial ability following stroke [39]. However, a current meta-analysis indicates that bilateral tDCS is inferior to unilateral tDCS for improving motor learning in stroke patients [74]. Interestingly, our data shows that only tDCS studies apply the bilateral stimulation in accordance with the assumed maladaptive role of the contralesional hemisphere [37]—with an anode over the ipsilesional and a cathode over the contralesional hemisphere [45, 50, 51, 57, 59]. In contrast, bilateral rTMS studies applied high-frequency protocols over either hemisphere [61, 65, 67].

The unilateral application of non-invasive brain stimulation induces, other than bilateral protocols, less consistent results across rTMS and tDCS/tACS/tsDCA studies. On the one hand, cathodal tDCS over the contralesional hemisphere induces large effects on gait, balance, and lower limb motor function [45]. On the other hand, low-frequency rTMS over this hemisphere had no effects [20, 62, 63, 66]. Interestingly, “facilitatory” stimulation of the contralesional hemisphere (contrary to its assumed maladaptive role) shows significant benefits with iTBS [64], but not with anodal tDCS [60]. Similarly, “facilitatory” stimulation of the ipsilesional hemisphere seems to be effective when using high-frequency rTMS [68], but not anodal tDCS [45, 47–49, 52, 58, 60]. Furthermore, cathodal tsDCS56 and the coupling of frontal anodal tDCS with parietal cathodal tDCS [53] did not induce relevant effects. Collectively, the data indicates that the application of “facilitatory” TMS protocols (high-frequency rTMS, iTBS) induces an improvement of the assessed parameters, regardless of the stimulated hemisphere. In contrast, the successful application of tDCS is strongly determined by the concept of interhemispheric competition after a stroke, as described above [27]. In this context, it must be pointed out that recent data challenges the traditional view of either “facilitatory” or “inhibitory” effects of specific rTMS and tDCS protocols. It has been shown that rTMS (1 Hz/5 Hz/15 Hz/20 Hz), iTBS/cTBS, anodal/cathodal tDCS may all lead to both increases and decreases the corticospinal excitability [27–30]. The direction and/or the amount of TMS- and tDCS-induced changes may be significantly determined by individual (sex, age, genetics, medication, pre-interventional MEP latency and amplitude, pre-interventional MT size), technical (stimulator type, neuro-navigation use, TMS pulse waveform) and methodological (target muscle and hemisphere, time after stimulation, time of day, behavioral context) factors [28, 75–77]. A better understanding of the impact of these variables on stimulation effects may optimize the therapeutic application of these methods. Therefore, future studies need to develop more individualized stimulation protocols in accordance with the current knowledge [28, 75–77].

Beside this, only a limited spectrum of stimulation intensities has been investigated in previous stroke studies [20, 45, 68]. The intensity of 2.5 mA has not been exceeded in any tDCS trial. The existing evidence indicates, however, that higher tDCS intensities (≥ 2.0 mA) are more effective than low-intensity stimulation (≤ 1.5 mA) (Fig. 4). Moreover, recent studies show that intensities up to 4 mA are safe, tolerable, and do not elicit any serious adverse effects [78, 79]. Therefore, tDCS intensities between 3.0 and 4.0 mA may have the potential to better support stroke recovery than present protocols. Another relevant issue is the individualization of tDCS intensity. In contrast to rTMS which is applied in every subject with a specific intensity that is determined relative to the individual motor threshold, tDCS is usually applied with the same, predefined intensity in all subjects. It is, therefore, an open question, whether individualization of tDCS intensity corresponding to the respective corticospinal excitability may increase stimulation effectiveness [80]. Regarding rTMS, reasonably good evidence exists for the effects of 1 Hz rTMS supporting walking, balance, and lower limb function in stroke cohorts. However, the available data indicates better effectiveness of 5–20 Hz rTMS protocols (Table 4). Thus, stimulation frequencies above 20 Hz may evoke even higher effects on motor recovery than present protocols. However, the risk of rTMS-induced seizures increases also with rTMS frequency. Therefore, the risk–benefit ration should be careful considered [81]. Up to now, stimulation protocols with frequencies of up to 50 Hz were successfully applied within the framework of neurorehabilitation [82]. Furthermore, most trials that were included in our meta-analysis applied rTMS with 90% of rMT. Only four studies applied another intensity (80% of active motor threshold [64], 100% [63, 65] and 120% [62] of resting motor threshold), and the available evidence did not show a superiority of any protocol.

A new way of tailoring TMS protocols is to consider the brain state of synchronized neuronal populations in the EEG at the time of stimulation. These EEG-triggered approaches may be informed by a series of post-hoc analyses of EEG features at the time of randomly applied TMS. In healthy subjects, stimulation effects on corticospinal excitability were less variable when the stimuli occurred at the optimum phase of beta frequency oscillations [83]. Along the same lines, the stimulation effects increased in both the resting and active motor system, when considering the oscillatory power of the beta-frequency band [84, 85]. Specifically, in both the resting brain85 and during voluntarily modulation [85], high and low beta-band activity decreased and increased corticospinal excitability, respectively. In addition, stimulation effects were modulated in a phase-dependent way along the oscillatory beta cycle and peaked with a diagonal shift of the highest stimulation response along the rising phase of the oscillatory cycle with increasing frequency [84]. Importantly, this phase-modulation was critically dependent on the precise temporal occurrence of the stimuli at a specific phase of the respective beta oscillatory cycle [86]. However, this high temporal precision could not be achieved in the past with EEG-triggered TMS approaches due to latencies between measurement and stimulation. Therefore, previous studies have applied EEG-controlled TMS on the basis of features that necessitated less temporal precision such as high and low oscillatory power levels in the beta band (16–22 Hz) [31, 32], and positive and negative peaks of the slow (< 1 Hz) [87] or alpha (8–12 Hz) [88, 89] oscillatory cycle. While beta power-dependent TMS induced robust increases of corticospinal excitability [31, 32], alpha peak-dependent findings were less consistent. Specifically, the alpha peak-dependent observations in a preselected group of participants with intrinsically high sensorimotor alpha power [88] could not be replicated, when the same approach of targeting the positive and negative peaks of the alpha cycle was applied in non-selected individuals [89]. Novel EEG-triggered approaches with integrated recording and stimulation devices and higher temporal precision may allow to repetitively target specific phases of higher frequency bands such as the oscillatory beta-band that have determined corticospinal excitability in previous post-hoc studies [83–86]. Moreover, such EEG-triggered approaches need also to be investigated in patient populations, e.g., following stroke [90], to explore their clinical utility under pathophysiological conditions.

### Stimulated area-dependent effect

It is an open question, how much influence the stimulation location has on gait, balance, and lower limb motor function after stroke. Most analyzed studies stimulated the primary motor cortex. Beside this, cerebellum, supplementary motor area and spinal cord were also targeted in a few trials. However, a direct comparison of these areas is difficult because of numerous additional variables, such as different stimulation protocols, stimulated hemispheres, patient cohorts etc. Future studies need to create larger evidence for the application of non-invasive stimulation of areas other than the primary motor cortex. The cerebellum is a highly promising candidate in this regard. The available data indicates that the cerebellum is, similar to M1, critically involved in motor learning, but the mechanisms underlying cerebellar stimulation differ from those related to M1 stimulation. Specifically, the cerebellum is more linked to predictions about the consequences of movement than to direct motor commands [91]. Moreover, “cerebellar inhibition” (i.e., the inhibitory tone of the cerebellum over M1 via the thalamus) seems to play a key role during error-based motor learning, which is differently involved during early and late skill learning [91]. A current experiment demonstrates that preconditioning cerebellar stimulation improves not only the performance during the subsequent learning phase of visuo-moto adaptation tasks, but also induces a sustained improvement in the re-adaptation of the recently learned skill [92]. This observation is important for neurorehabilitation. It is an open question, however, whether and to which extend a stroke-induced damage of cortical motor areas may be compensated by cerebellar structures. In general, “cerebellar reserve” refers to the capacity of this area to compensate for tissue damage or loss of function following different etiologies [93]. Thus, it is plausible that cerebral stimulation may be a good alternative for patients that suffer from extensive cortical damage. Furthermore, it is conceivable that other cortical (such as the inferior parietal and frontal cortex) and subcortical (such as basal ganglia, thalamus, and hippocampus) regions and the brainstem are suitable for the application of non-invasive stimulation techniques for supporting gait and balance recovery [9, 13, 14]. Furthermore, the development of innovative technical devices enables modulating brain regions that could insufficiently be targeted by conventional stimulation equipment [26, 94, 95]. Double-cone coils, for example, are larger version of the standard figure-8 coils, and have two circular windings angled towards the subject’s head. Such double-cone coils are less focal but stimulate deeper brain areas than conventional figure-8 coils and may thus be beneficial for targeting the leg motor area, medial prefrontal cortex, cingulate, insula, and cerebellum [26, 94]. Similarly, other coil design (H, crown, stretched C-core, triple halo) have also the potential to modulate deeper brain areas than conventional figure-8 coils [94, 95], and their effectiveness in supporting gait, balance and lower limb function need to be investigated in future studies.

### Patient characteristic-dependent effects

The studies included in our meta-analysis demonstrate an inconsistency of subjects regarding time since stroke, stroke etiology and lesion location. All these factors may hamper the interpretation of the results. Future studies should devote more attention to these important aspects. Studies investigating non-invasive brain stimulation for improving hand motor recovery could detect that lesion location may determine the effectiveness of the treatment [96, 97]. Fifteen sessions of 1 Hz rTMS over the contralesional primary motor cortex, for example, supported motor function of the affected hand only in patients with lesion of the dominant hemisphere. Patients with an injury of the non-dominant hemisphere did not profit from the intervention.98 Similarly, a single session of 10 Hz rTMS over the ipsilesional primary motor cortex significantly improved motor function of the affected upper limb in patients with a subcortical lesion. In contrast, no changes were detected in patients with cortical involvement [96]. Furthermore, a fMRI study detected different activation patterns during active movement of the affected lower limb in patients with subcortical and cortical stroke [98]. The data revealed similar activation patterns in patients with subcortical lesion and healthy controls with the recruitment of the contralateral primary motor cortex, supplementary motor area, and bilateral somatosensory area. In contrast patients with cortical stroke and brainstem stroke showed reduced cortical recruitment [98]. Thus, it is conceivable that different stimulation protocols may be beneficial, depending on the lesion location.

## Strength and limitations

To our knowledge, this is the first meta-analysis that compared the effectiveness of different non-invasive stimulation protocols in supporting gait, balance, and lower limb motor function in stroke subjects. An important strength is that we included and analyzed more studies than previously articles to this topic [69–72]. The main weakness is the inconsistency of analyzed studies with regard to the included patients (different time period since the incident, different stroke etiology and location), methodological approach (different numbers of intervention-sessions, different evaluation schedules), interventions (different stimulation protocols, different stimulation duration, different stimulated areas) and outcomes (more than twenty different assessments). Further weaknesses are the methodological limitations of the analyzed studies: (1) the absence of concealed allocation, (2) the absence of therapist blinding and (3) the absence of intention to treat analysis (Table 2). This may hamper the interpretation of the results.

## Conclusions

This systematic review and meta-analysis show that certain types of non-invasive neuromodulation are effective in improving gait, balance, and lower limb motor function in stroke survivors. Available data indicates that (1) tDCS/tACS/tsDCS is more effective than rTMS, and that (2) bilateral stimulation is more effective than unilateral stimulation. However, more research is needed to maximize the effectiveness of existing protocols by optimizing stimulation dosage, intensity, and duration, by considering the brain state with EEG-triggered interventions, and by better characterizing the targeted stroke cohorts that may benefit.

## Data Availability

The datasets generated during and/or analyzed during the current study are available from the corresponding author on reasonable request.

## Abbreviations

cTBS: Continuous theta burst stimulation
EEG: Electroencephalography
Hz: Hertz
iTBS: Intermittent theta burst stimulation
mA: Milliampere
MEP: Motor evoked potential
MR: Magnetic resonance imaging
ms: Millisecond
M1: Primary motor cortex
na: Not available/not applicable
PEDro: Physiotherapy Evidence Database
PRISMA: Preferred Reporting Items for Systematic Reviews and Meta-Analysis
tACS: Transcranial alternating current stimulation
tDCS: Transcranial direct current stimulation
tsDCS: Trans spinal direct current stimulation
rMT: Resting motor threshold
(r)TMS: (Repetitive) transcranial magnetic stimulation
